# Serum lactate level predicts 6-months mortality in patients with hepatitis B virus-related decompensated cirrhosis: a retrospective study

**DOI:** 10.1017/S0950268820003143

**Published:** 2021-01-05

**Authors:** Yuan Nie, Lin-Xiang Liu, Tao Chen, Yue Zhang, Xuan Zhu

**Affiliations:** 1 Department of Gastroenterology, The First Affiliated Hospital of Nanchang University, Nanchang, Jiangxi, China; 2 Department of Gastroenterology, Fuzhou First People's Hospital, Fuzhou, Jiangxi, China

**Keywords:** Decompensated cirrhosis, HBV, lactate, prognosis

## Abstract

The prediction of prognosis is an important part of management in hepatitis B virus (HBV)-related decompensated cirrhosis patients with high long-term mortality. Lactate is a known predictor of outcome in critically ill patients. The aim of this study was to assess the prognostic value of lactate in HBV-related decompensated cirrhosis patients. We performed a single-centre, observational, retrospective study of 405 HBV-related decompensated cirrhosis patients. Individuals were evaluated within 24 h after admission and the primary outcome was evaluated at 6-months. Multivariable analyses were used to determine whether lactate was independently associated with the prognosis of HBV-related decompensated cirrhosis patients. The area under the ROC (AUROC) was calculated to assess the predictive accuracy compared with existing scores. Serum lactate level was significantly higher in non-surviving patients than in surviving patients. Multivariable analyses demonstrated that lactate was an independent risk factor of 6-months mortality (odds ratio: 2.076, *P* < 0.001). Receiver operating characteristic (ROC) curves were drawn to evaluate the discriminative ability of lactate for 6-months mortality (AUROC: 0.716, *P* < 0.001). Based on our patient cohort, the new scores (Model For End-Stage Liver Disease (MELD) + lactate score, Child–Pugh + lactate score) had good accuracy for predicting 6-months mortality (AUROC = 0.769, *P* < 0.001; AUROC = 0.766, *P* < 0.001). Additionally, the performance of the new scores was superior to those of existing scores (all *P* < 0.001). Serum lactate at admission may be useful for predicting 6-months mortality in HBV-related decompensated cirrhosis patients, and the predictive value of the MELD score and Child–Pugh score was improved by adjusting lactate. Serum lactate should be part of the rapid diagnosis and initiation of therapy to improve clinical outcome.

## Introduction

Liver cirrhosis has high morbidity and mortality and leads to 1.03 million deaths per year, making it the 14th most common cause of death worldwide [1]. In China, survival with cirrhosis is even less optimistic because many hepatitis B virus (HBV)-infected patients progress to cirrhosis without effective antiviral therapy [2, 3]. However, compensated cirrhosis is not easy to detect, and most patients are diagnosed with decompensated cirrhosis in the hospital because of cirrhosis complications such as ascites, gastrointestinal haemorrhage, hepatic encephalopathy (HE) and hepatorenal syndrome (HRS). The 1-year mortality of liver cirrhosis varies greatly, from 1% to 57%, according to the complications of HBV-related decompensated cirrhosis [4]. Decompensated cirrhosis carries a poor prognosis because the median survival time is about 2 years, and it imposes a heavy burden on health-care costs, mainly due to the need for repeated hospital admissions [5]. Although liver transplantation can significantly improve survival, it is not widely applied due to shortcomings in liver sources, costs and technology [6]. Therefore, it is necessary to use prognostic models to identify high-risk patients. Severity scores have been developed for HBV-related decompensated cirrhosis patients admitted to the hospital or intensive care unit (ICU) based on combinations of prognostic indicators [7–11]. The Child–Pugh and Model For End-Stage Liver Disease (MELD) scores have been widely used to predict the outcomes of cirrhotic patients, but they have obvious deficiencies [12–15]. Therefore, a simple and practicable parameter is necessary to boost the predictive efficiency of scores and to guide the choice of therapeutic measures.

Elevated lactate levels may be due to anaerobic metabolism and oxidative stress, in which case it is a marker of tissue hypoxia, or metabolic changes due to stress reactions by the release of epinephrine [16]. A previous study indicated that higher lactate levels and reduced lactate clearance are associated with mortality in critically ill patients, especially in septic patients [17, 18]. Sepsis and infections are common reasons for hospital admission in decompensated cirrhosis patients in China and are associated with poor outcomes [19]. Lactate levels may be a useful tool for assessing the severity of disease in critically ill patients with cirrhosis admitted to the ICU. In a recent report, lactate levels and clearance were independently associated with short-term mortality in critically ill patients with liver cirrhosis, and the lactate-adjusted score was significantly better than the existing scoring system [11]. However, the study populations have been dominated by Caucasians in Europe and the USA, and the most common cause of liver cirrhosis is alcohol. In the national population of cirrhosis caused by hepatitis viral, whether lactate levels can also serve as a prognostic indicator for decompensated cirrhosis patients is still unknown. Previous studies have focused on short-term deaths, such as 1, 3-months, and there have been fewer reports on long-term deaths. The aim of this study was to assess the prognostic performance of lactate levels for 6-months mortality in HBV-related decompensated cirrhosis patients admitted to the hospital in China to guide clinical practice.

## Materials and methods

### Study subjects

The study cohort included all patients aged ⩾18 years with cirrhosis of the liver who were hospitalised due to HBV-related decompensated cirrhosis. Refusal to give consent, age <18 years, pregnancy, cerebrovascular disease, cardiovascular disease, hematologic disorders and renal failure were exclusion criteria. All patients were treated following accepted recommendations and guidelines after admission to the hospital, and they were followed up until death or 6 months [20, 21]. The study protocol was approved by the institutional ethics committee of the First Affiliated Hospital of Nanchang University (No. 2013-0102). Written informed consent was obtained from all the study participants.

### Definitions

Patients with chronic HBV infection were confirmed by the detection of hepatitis B surface antigen positivity for more than 6 months. The decompensated cirrhosis was diagnosed by the presence of ascites, HE, HRS and/or variceal bleeding at the time of the study. HE and acute-on-chronic liver failure (ACLF) were diagnosed according to West Haven criteria, well-established criteria defined by the chronic liver failure consortium (CLIF-C). HRS and ascites were diagnosed using the criteria proposed by the International Ascites Club and American Association for the Study of Liver Disease [22–24]. The Child–Pugh score was calculated according to total bilirubin (TBIL), albumin, international normalised ratio (INR), ascites status and degree of HE. The MELD score was calculated using the formula: 3.78 × Ln (TBIL μmol/l) + 11.2 × Ln (INR) + 9.57 × Ln (creatinine μmol/l) + 6.43 × (constant for liver disease aetiology = 0, if cholestatic or alcoholic, otherwise = 1). New scores (MELD + lactate score, Child–Pugh + lactate score) were created by adding lactate to the MELD score and Child–Pugh score. For example, if the Child–Pugh score of a patient is 10 and the lactate level value is 2, the new Child–Pugh score is 12; if the MELD score of a patient is 15 and the lactate level value is 3, the new MELD score is 16.

### Study protocol

Patients were enrolled in the study when they were hospitalised with HBV-related decompensated cirrhosis. During the index hospitalisation, data were collected and compiled regarding the demographic profile, history, clinical features, presence of other comorbidities, aetiology of the cirrhosis, type of decompensation, number of complications and blood laboratory parameters at admission (white blood cell count (WBC), platelets (PLT), serum sodium (Na), creatinine (Cr), serum urea, alkaline phosphatase, *γ*-glutamyl transpeptidase (GGT) from venous blood; lactate, oxygenation index (PaO_2_/FiO_2_) from arterial blood) before treatment. Lactate was measured from the first radial or femoral artery within 24 h of admission. Blood samples were collected in heparinised blood gas syringes and measured by blood gas analyser (ABL 800 Series; Radiometer, Denmark) using the enzyme electrode method at 37 °C. Patients were followed for 6 months to calculate survival using liver clinic records, institutional medical records or telephone conversations. Patients with incomplete follow-up were not included in the final analysis.

### Statistical analysis

Statistical analyses were performed using SPSS software version 22.0 (SPSS Inc., Chicago, IL, USA), and receiver operating characteristic (ROC) analysis was done by using MedCalc statistical software version 15.2.1 (MedCalc, Ostend, Belgium). Continuous and categorical variables were initially described as median (interquartile range) and frequency (percentage (%)), respectively. Continuous variables were compared using the Mann–Whitney *U* test, and categorical variables were compared using the *χ*^2^ test or Fisher's exact test. Multivariable analysis was employed to demonstrate the independent predictors for the mortality rate of patients with decompensated cirrhosis. All variables that were found to be associated with mortality (*P* < 0.100) were included as candidate variables in a forward stepwise logistic regression analysis to identify independent predictors for the prognosis of decompensated cirrhosis patients. The diagnostic accuracy of prognostic variables was examined by ROC analysis with comparisons between areas under the ROC curves (AUROC), done with the De Long test. Sensitivity and specificity were determined using the cut-off point with the highest Youden index (sensitivity + specificity − 1). All statistical tests were two-sided, and a value of *P* < 0.050 was considered statistically significant.

## Results

### Demographic data

A total of 405 patients were hospitalised with HBV-related decompensated cirrhosis from January 2013 to December 2019 in this retrospective study. Patient age ranged from 25 to 86 years (median: 53.5 years). The majority of the patients were male (302/405, 74.6%). The most common decompensation events responsible for hospitalisation were gastrointestinal haemorrhage (61.2%), infection (23.2%), HE (8.1%) and ascites (7.4%). The average length of hospital stay was 10 (8–12) days. Sixty-eight patients (16.7%) received treatment in the ICU, and 337 (83.2%) patients received treatment in the general ward. A total of 298 patients had been followed up to 6 months, including 84 patients who died. The causes of death were as follows: 15 (17.9%) from respiratory failure, 39 (46.4%) patients from haemorrhagic shock, nine (10.7%) patients from HE, eight (9.5%) patients from infectious shock, five (5.9%) patients from HRS, four (4.8%) patients from liver failure and four (4.8%) patients from uncertain causes. The baseline characteristics of this cohort are presented in Table 1.

**Table 1.** Patients' characteristics of HBV-related decompensated cirrhosis cohort

Variable	Decompensated patients with cirrhosis (*n* = 405)	Survivors (*n* = 214)	Non-survivors (*n* = 84)	*P* value
Sex (man), *n* (%)	302 (74.6%)	151 (70.6%)	62 (73.8%)	0.576
Age, median (IQR)	53.5 (46–63.75)	54 (47–63)	56 (51.5–67)	0.357
Hospitalisation days, median (IQR)	10 (6–12)	8(5.5–11.5)	14(9–19.5)	**<0**.**001**
Intensive care unit, *n* (%)	68 (16.7%)	42 (19.7%)	28 (33.3%)	**0**.**012**
Cause of hospitalisation, *n* (%)				0.077
Ascites	30 (7.4%)	1 (0.5%)	2 (2.4%)	
Gastrointestinal haemorrhage	248 (61.2%)	179 (83.6%)	67 (79.8%)	
Hepatic encephalopathy	33 (8.1%)	26 (12.1%)	7 (8.3%)	
Infection	94 (23.2%)	8 (3.7%)	8 (9.5%)	
Ascites degree, *n* (%)				0.086
No ascites	165 (40.7%)	92 (32.4%)	33 (39.3%)	
1st-degree ascites	109 (26.9%)	61 (21.5%)	17 (20.3%)	
2nd-degree ascites	80 (19.8%)	34 (12.0%)	14 (16.7%)	
3rd-degree ascites	51 (12.6%)	27 (9.5%)	20 (23.8%)	
Acute renal failure, *n* (%)	14 (3.5%)	7 (2.5%)	5 (6.0%)	0.289
Acute chronic liver failure, *n* (%)	19 (4.7%)			
Hepatocellular carcinoma, *n* (%)	36 (8.8%)	16 (5.6%)	17 (20.2%)	**0**.**002**
Therapy, *n* (%)				**<0**.**001**
Vasopressor support	127 (31.3%)	85 (29.9%)	49 (58.3%)	
Mechanical ventilation	27 (6.6%)	3 (1.0%)	16 (19.0%)	
Renal replacement therapy	2 (0.49%)	1 (0.5%)	1 (1.2%)	

IQR, interquartile range.

The Bolded figures indicate statistically significant comparison (P < 0.05).

### Association between mortality and clinical or laboratory characteristics

The clinical and laboratory characteristics of these patients are listed in Table 2. HBV-related decompensated cirrhosis patients were divided into non-surviving (*n* = 84) and surviving groups (*n* = 214) according to 6-month survival outcomes. The majority of non-survivors had been graded higher, as reflected by ALT, AST, bilirubin, GGT, Cr, INR, PT, WBC, lactate, Child–Pugh score and MELD score. However, albumin levels were lower in non-survivors. No significant differences in PLT, serum Na, MAP or PO_2_/FiO_2_ were detected.

**Table 2.** The association between clinical or laboratory characteristics and mortality in HBV-related decompensated cirrhosis patients

Parameter	Survivors (*n* = 214)	Non-survivors (*n* = 84)	*P* value
ALT, IU/l	23 (17–36)	26 (15–56)	**0**.**040**
AST, IU/l	35.5 (26–53)	58 (36–168)	**<0**.**001**
Albumin, g/l	29.1 (25.925–32.1)	25.7 (22.8–29.5)	**<0**.**001**
Bilirubin, mmol/l	21.9 (14.3–37.475)	29.5 (19.6–57.2)	**0**.**004**
GGT, IU/l	21 (13–48)	37 (17–106)	**0**.**003**
Creatinine, mmol/l	71.45 (57.7–86.075)	91.9 (63.4–132.6)	**<0**.**001**
INR	1.31 (1.19–1.487)	1.4 (1.24–1.76)	**<0**.**001**
PT	14.7 (13.025–16.4)	15.4 (13.7–19.9)	**<0**.**001**
Platelets, 10^9^/l	64 (41–92.75)	70 (37–109)	0.419
WBC, 10^9^/l	6.295 (3.805–9.432)	7.75 (4.42–13.57)	**0**.**003**
Na, mmol/l	138.6 (136–141.15)	138.5 (135–142)	0.967
MAP, mmHg	82.667 (79–88)	83.667 (77.667–89.33)	0.909
PO_2_/FiO_2_, mmHg	411 (349.25–480.5)	391 (301–452)	0.060
Lactate, mmol/l	1.7 (1.3–2.4)	3 (1.6–4.75)	**<0**.**001**
Child–Pugh score	8 (7–9)	9.5 (8–11)	**<0**.**001**
MELD score	10 (9–14)	14 (11–19.5)	**<0**.**001**

ALT, alanine aminotransferase; AST, aspartate aminotransferase; GGT, *γ*-glutamyl transpeptidase; INR, international normalised ratio; PT, prothrombin time; WBC, white blood cell count; MAP, mean artery pressure; PO_2_/FiO_2_, oxygenation index; MELD, model for end-stage liver disease.

Data are expressed as median (interquartile range).

The Bolded figures indicate statistically significant comparison (P < 0.05).

The results of *χ*^2^ test had shown that >50 years of age, third-degree ascites, hepatocellular carcinoma (HCC) were risk factors for 6-months mortality (OR 1.852 (1.052–3.261); *P* = 0.033; OR 2.227 (1.107–4.483); *P* = 0.025; OR 1.977 (1.386–2.821); *P* < 0.001). Then, multivariable logistic regression analysis identified that >50 years of age, HCC, GGT and lactate were risk factors for 6-months mortality in patients with HBV-related decompensated cirrhosis patients (*P* < 0.05) (Table 3). No significant effect was noted for sex, cause of hospitalisation, acute renal injury, PLT, serum Na, mean arterial pressure or PaO_2_/FiO_2_ (*P* > 0.05).

**Table 3.** Multivariable analyses of risk factors associated with mortality for 6-months days

Variable	Multivariate	
	OR (95% CI)	*P* value
Age		**0**.**002**
⩽50	Reference	
>50	2.774 (1.082–4.901)	
HCC	5.932 (2.155–16.873)	**<0**.**001**
GGT	1.153 (1.021–1.330)	**0**.**012**
Lactate	2.076 (1.148–2.312)	**<0**.**001**
Child–Pugh score	1.767 (1.099–2.560)	**<0**.**001**
MELD score	1.905 (1.114–2.610)	**<0**.**001**

CI, confidence interval; OR, odds ratio; HCC, hepatocellular carcinoma; GGT, *γ*-glutamyl transpeptidase; MELD, model for end-stage liver disease.

The Bolded figures indicate statistically significant comparison (P < 0.05).

### Predictive value for 6-months mortality in HBV-related decompensated cirrhosis patients

**Fig. 1.**
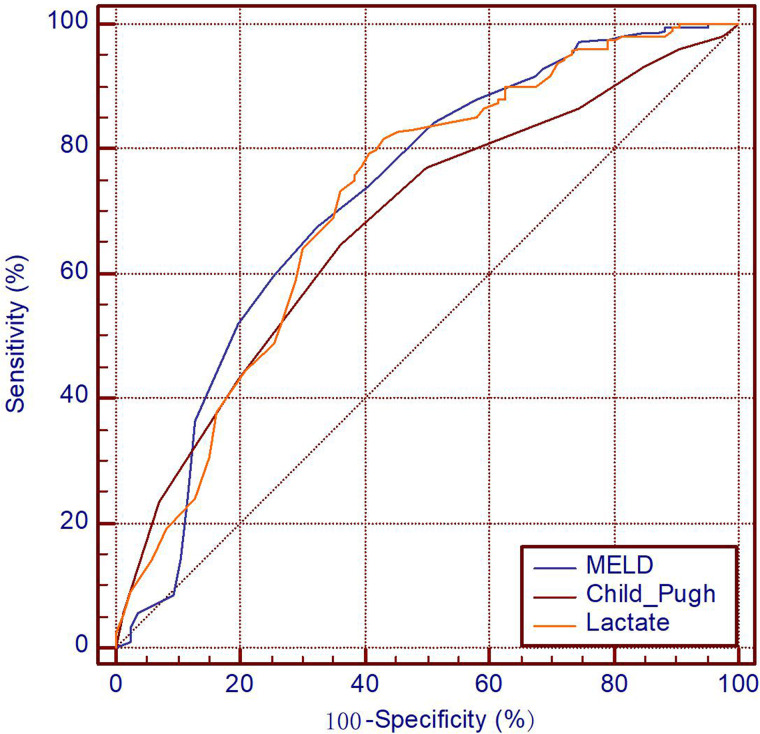
Receiver operating characteristic curves of lactate, MELD score, Child–Pugh score. MELD, model for end-stage liver disease score; Child–Pugh, Child–Pugh score.

Figure 1 shows the performance analysis of the discriminative accuracy of lactate for 6-months mortality with AUROC of 0.716 (95% CI 0.649–0.784, *P* < 0.001). The AUROC of MELD score and Child–Pugh score were 0.723 (95% CI 0.654–0.791, *P* < 0.001) and 0.679 (95% CI 0.613–0.744, *P* < 0.001), respectively. The ROC curves and comparison of prognostic scores are shown in Figure 1 and Table 4, respectively.

**Table 4.** Comparison of prognostic scores in predicting 6-months mortality

Prognostic score	ROC area	*P* value	Cut-off point	Sensitivity (%)	Specificity (%)	PLV	NLV
Lactate, mmol/l	0.699	<0.0001	2.6	79.16	56.98	1.84	0.37
MELD score	0.723	<0.0001	12	67.62	67.44	2.08	0.48
Child–Pugh score	0.679	<0.0001	8	64.76	63.95	1.80	0.55

MELD, model for end-stage liver disease; PLV, positive likelihood ratio; NLV, negative likelihood ratio.

### Predictive value of MELD score and Child–Pugh score is improved by adjusting lactate

**Fig. 2.**
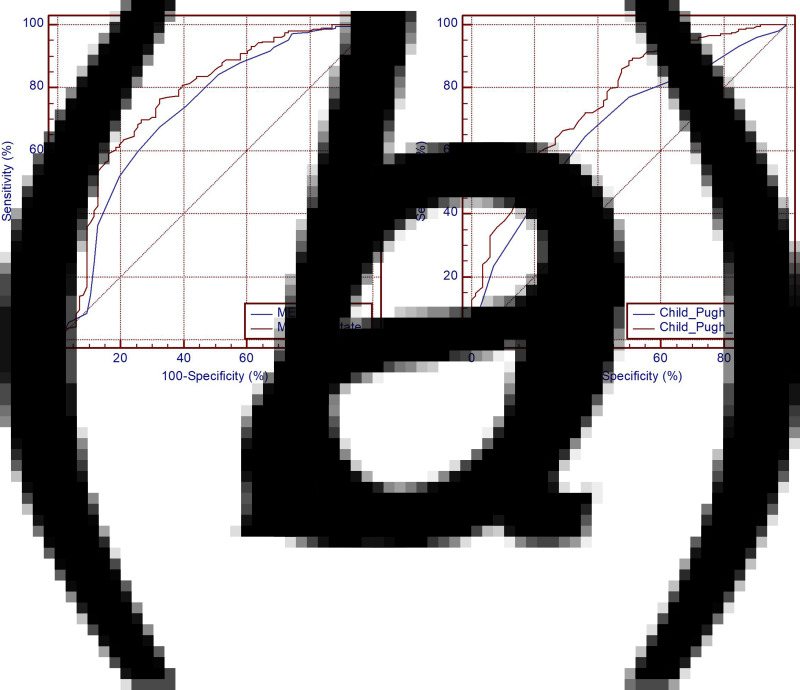
Comparing the receiver operating characteristic curves of the scores. MELD, model for end-stage liver disease score; Child–Pugh, Child–Pugh score. (A) ROC for MELD score *vs*. MELD + lactate score; (B) ROC for Child–Pugh score *vs*. Child–Pugh + lactate score.

To improve the predictive value, new scores were established. In the same dataset, an analysis of AUROC at 6-months mortality showed that MELD + lactate score and Child–Pugh + lactate score were superior to MELD score and Child–Pugh score, respectively (difference between areas = 0.045, 95% CI 0.017–0.073, *Z* = 3.191, *P* = 0.001; difference between areas = 0.087, 95% CI 0.043–0.131, *Z* = 3.874, *P* < 0.001). The ROC curve and comparison of prognostic scores are shown in Figure 2 and Table 5, respectively.

**Table 5.** Comparison of prognostic scores in predicting 6-months mortality

Prognostic score	ROC area	*P* value	Cut-off point	Sensitivity (%)	Specificity (%)	PLV	NLV
MELD + lactate	0.769	<0.0001	15.8	76.67	67.44	2.35	0.35
Child–Pugh + lactate	0.766	<0.0001	10	59.52	79.07	2.84	0.51

MELD, model for end-stage liver disease; PLV, positive likelihood ratio; NLV, negative likelihood ratio.

## Discussion

Assessment of prognosis, especially in patients with cirrhosis at the hospital, is of crucial importance to guide therapeutic measures [25]. To our knowledge, this is the most comprehensive study to evaluate the diagnostic accuracy of MELD score and Child–Pugh score in patients with liver cirrhosis [13, 26]. As we expected, MELD score and Child–Pugh score were associated with 6-months prognosis in the univariate analysis, and multivariate logistic regression also identified that albumin and Cr, which are parameters of the MELD score, as predictive factors for 6-months mortality. The ROCs of the MELD score and Child–Pugh score were drawn, and the AUROC of those scores was 0.723 and 0.679, respectively. However, they have had obvious deficiencies in previous studies. First, ascites and HE, included in the Child–Pugh score, are subjective and may vary according to the physicians' judgment and the use of diuretics and lactulose. Second, INR, which is one component of both Child–Pugh and MELD scores, does not sufficiently reflect coagulopathy and consequently liver function in liver cirrhosis [15]. Third, there is an interlaboratory variation in INR value [14]. Most studies on the MELD score and Child–Pugh score have been done in Western countries, where the main cause of cirrhosis is alcohol [27–29]. However, in this study, HBV-related cirrhosis and gastrointestinal bleeding, which account for most of the cirrhotic population in China, may reduce the prediction efficiency of the score. Therefore, it is meaningful to find a simple and practicable indicator to increase the predictive efficiency of the score in HBV-related decompensated cirrhosis patients to guide clinical treatment.

Serum lactate levels are closely associated with tissue hypoxia and anaerobic metabolism, and are additionally strongly correlated with death [18, 30]. Various studies provide considerable evidence for the prognostic value of lactate in critically ill patients [31, 32]. A study had found that the main metabolites (including lactate) are high in ACLF patients [33]. Lactate level is increased in non-surviving patients with decompensated cirrhosis by 1H nuclear magnetic resonance spectroscopy and reversed-phase ultra-performance liquid chromatography coupled to time-of-flight mass spectrometry [34]. Hyperlactatemia at the time of admission to the hospital has been proposed to be a potential marker for postoperative morbidity and mortality with high sensitivity and specificity for adverse effects [35]. Serum lactate levels are currently used in risk stratification of patients with sepsis, trauma and pulmonary embolism [36–38]. Several studies have revealed that the lactate level has been associated with mortality among patients admitted to the ICU in cirrhotic patients. A multinational study had revealed that lactate appropriately reflected the severity of disease and organ failure and was independently associated with short-term mortality in critically ill patients with liver cirrhosis after nearly 1 year of follow-up, and the performance of CLIF-C ACLF score was significantly improved by adjusting lactate [11]. Tas's multinational research has revealed that the lactate level and Acute Physiology and Chronic Health Evaluation (APACHE II) score are the two best predictive factors of short-term mortality in cirrhotic elderly patients admitted to the ICU [39]. However, their study populations were dominated by Caucasians in Europe and the USA, and most cases of liver cirrhosis in those populations are caused by alcohol. In a study of 949 critically ill cirrhotic patients with acute respiratory failure, a new score (ARF-CLIF-SOFA) contained serum lactate and had better accuracy for predicting 30-days, 90-days and 1-year mortality compared to the MELD score and chronic liver failure-sequential organ failure assessment (CLIF-SOFA) score [40]. Serum lactate levels were associated with a higher mortality rate in critically ill patients with cirrhosis with acute kidney injury [41]. However, their study did not clearly reveal that lactate levels were associated with prognosis in decompensated cirrhosis patients in China. Therefore, we designed and completed the study reported here. Our study was a single-centre, large sample, observational retrospective analysis that evaluated simple laboratory parameters as predictors of mortality in HBV-related decompensated cirrhosis patients.

This paper aimed to validate the predictive value of lactate for 6-month mortality in HBV-related decompensated cirrhosis admitted to the hospital. First, we found that lactate was significantly higher in non-surviving patients than in surviving patients (Table 2) and served an independent risk factor for long-term mortality in single variable and multivariable analysis (Table 3). More importantly, our results indicated that lactate was able to predict long-term mortality in decompensated cirrhosis patients (Table 4 and Fig. 1) and that the AUROC of the new scores (MELD + lactate score, Child–Pugh + lactate score) were higher than those of the MELD score and Child–Pugh score, respectively, based on the same data (Table 4 and Fig. 1). Our findings demonstrate that lactate is also a good and independent predictor of long-term outcomes in HBV-related decompensated cirrhosis patients of the Chinese population.

The underlying mechanisms by which serum lactate can predict the prognosis of patients with HBV-related decompensated cirrhosis are not well defined. Lactate is considered the main end-product of anaerobic glycolysis (Embden–Meyerhof pathway), where nicotinamide adenine dinucleotide is regenerated by the glycolytic lactate dehydrogenase system upon redox-coupled reduction of pyruvate to lactate [42]. Approximately 1500 mmol of lactate is produced daily in the human body, primarily by highly glycolytic tissues [43]. Traditionally, lactate has been deemed a marker for tissue hypoxia, and the generation and consumption of lactate are strictly balanced in physiological conditions. Hyperlactatemia is usually the result of either increased lactate production or reduced consumption [44]. The liver is responsible for up to 70% of whole-body lactate clearance, especially hepatic impairment, which is associated with increased lactate levels due to impaired mitochondrial oxidation [45]. Another source of hyperlactatemia is increased lactate production, which is related to microcirculatory oxygen imbalance [43]. Patients with unstable haemodynamics demonstrate increased ketone/pyruvate ratios and decreased arterial ketone body ratios related to anaerobic production. Previous studies revealed that in the absence of circulatory delivery relative to systemic metabolic demand, microcirculatory processes hampering oxygen utilisation at the tissue level may raise lactate levels [46]. Circulatory failure is also considered a complication related to mortality in critically ill cirrhotic patients [47]. Furthermore, mechanisms other than tissue hypoxia can account for hyperlactatemia, such as drugs and intoxicants and pyruvate dehydrogenase dysfunction [48]. Hence, we assume that the relationship between blood lactate and prognosis of HBV-related decompensated cirrhosis patients is the result of comprehensive effects on metabolism and microcirculation.

This study has several limitations. First, as a single-centre, retrospective cohort study, some patients were lost to follow-up, which may have resulted in selection bias. The findings need to be confirmed in large, multicentre studies. Second, we were not able to evaluate the predictive role of dynamic changes of lactate, as the long-term changes in serum lactate and lactate clearance were not routinely measured in clinical practice. Finally, organ failure-related scores, such as SOFA score and CLIF-C ACLF score, were not calculated, and the correlation between organ failure and lactic acid was not analysed.

In conclusion, many factors may be useful as a predictor of mortality in the hospital in patients with HBV-related decompensated cirrhosis, including MELD score and Child–Pugh score. Our results indicate that the initial lactate level strongly and independently predicts long-term outcomes. In terms of prognostic value, lactate levels demonstrate a similar discriminatory power as the MELD score and Child–Pugh score, and the predictive efficiency of those existing scores is elevated by adding lactate. From a clinical perspective, using lactate might be more convenient, as fast diagnosis and initiation of therapy are essential in reducing mortality.

## Data

The data that support the findings of this study are available from the corresponding author, X.Z., upon reasonable request
